# Antitumoral effect of Ocoxin on acute myeloid leukemia

**DOI:** 10.18632/oncotarget.6862

**Published:** 2016-01-09

**Authors:** Elena Díaz-Rodríguez, Susana Hernández-García, Eduardo Sanz, Atanasio Pandiella

**Affiliations:** ^1^ Instituto de Biología Molecular y Celular del Cáncer CSIC-Universidad de Salamanca, Salamanca, Spain; ^2^ Catalysis, S.L., Madrid, Spain

**Keywords:** acute myeloid leukemia, antioxidants, cell cycle, p27

## Abstract

Acute myeloid leukemia (AML) is a heterogeneous hematological malignancy whose incidence is growing in developed countries. In the relapse setting, very limited therapeutic options are available and in most cases only palliative care can be offered to patients. The effect of a composite formulation that contains several antioxidants, Ocoxin Oral solution (OOS), was tested in this condition. When analyzed *in vitro*, OOS exhibited anti-AML action that was both time and dose dependent. *In vivo* OOS induced a ralentization of tumor growth that was due to a decrease in cell proliferation. Such effect could, at least partially, be due to an increase in the cell cycle inhibitor p27, although other cell cycle proteins seemed to be altered. Besides, OOS induced an immunomodulatory effect through the induction of IL6. When tested in combination with other therapeutic agents normally used in the treatment of AML patients, OOS demonstrated a higher antiproliferative action, suggesting that it may be used in combination with those standard of care treatments to potentiate their antiproliferative action in the AML clinic.

## INTRODUCTION

Acute myeloid leukemia (AML) is a complex and heterogeneous hematological malignancy in which a multipotent hematopoietic stem cell or progenitor cell will be transformed to a leukemia stem cell as a result of the accumulation of genomic alterations [1]. AML is a disease predominantly of adults with a median age at diagnosis of 69 years [2, 3]. Probably due to the general population aging or to the fact that AML can also appear as a second cancer in patients previously treated with chemo or radiotherapy [4, 5], its incidence is rising in developed countries. Despite recent advances in its diagnosis and treatment, up to 75% of the patients will die of this disease [6]. On the other hand, for patients with relapsed AML, treatment options are very limited and in most of the cases only palliative care can be offered.

In the past few years, multiple studies have demonstrated that the co-administration of combination of various antioxidants with conventional chemotherapy or radiotherapy results in significant antitumor synergism and an inhibition of the toxicity of conventional therapy [7, 8]. Thus, while the pure compounds are not available for use in the oncology clinic, commercial formulations which include some of these molecules offer the possibility to incorporate them easily and safely to the treatments commonly used to treat certain neoplasias [9]. Such is the case of Ocoxin Oral Solution (OOS), an oral nutritional supplement that includes recognized anti-cancer compounds. OOS contains green tea polyphenols such as epigallocatechin 3-gallate, vitamin B6 and vitamin C, three products whose anticancer action has been proven in several studies [8, 10]. OOS also contains cinnamic acid, a compound which may inhibit tumor growth [11], as well as glycyrrhizinic acid, whose action as anti-inflammatory and immunomodulator has been reported [12].

OOS is being currently investigated in clinical trials as part of the treatment for several types of cancer, mainly associated with chemotherapy and radiotherapy. In these studies, an improvement in the quality of life of the patients has been reported [13]. Although the mechanism of action of OOS is largely unknown, a recent report has demonstrated the antitumoral action of OOS in preclinical models of HER2+ breast cancer [14]. In these models, OOS provoked cell cycle blockage by the upregulation of p27 and downregulation of cyclin D1 as well as an increase in apoptotic cell death. Moreover this formulation potentiated the action of lapatinib, a HER2 inhibitor used in breast cancer clinic [14].

Based on these previous results, the effects of OOS on preclinical models of AML were explored. Its potential antiproliferative action on several AML cell lines was initially tested *in vitro*. Later on, OOS effects *in vivo* were determined on a xenograft model in which the AML cell line HEL was used to generate the tumors. In that context, OOS reduced tumor growth in vivo. Moreover in this system cell cycle blockage was also linked to an increase in p27. Finally, combination studies aimed at evaluating if OOS could augment the efficacy of standard of care drugs used in AML clinic showed that this compound potentiates the antitumoral action of those drugs, giving the rationale to explore its clinical value on AML patients.

## RESULTS

### Effect of OOS on the proliferation of AML cell lines

**Figure 1:**
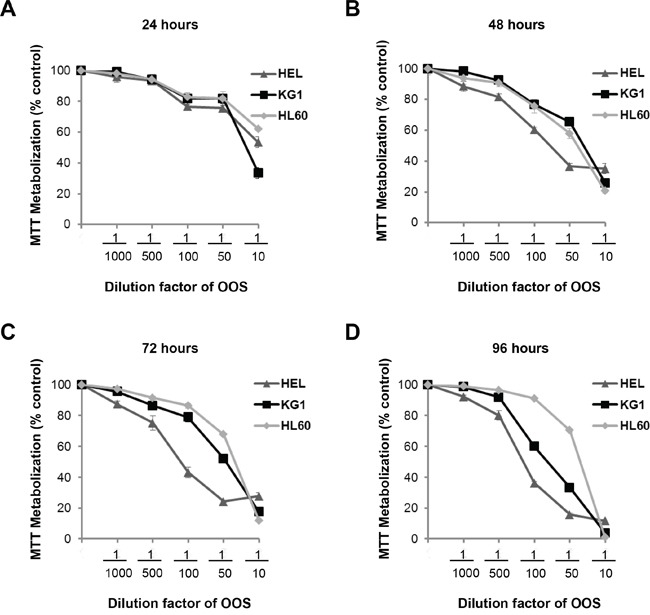
Efficacy of OOS on AML cell lines *in vitro* Dose-dependent effect of OOS on the proliferation of HEL, KG1 or HL60 AML cells was assessed *in vitro*. Cells were incubated with OOS at the indicated dilution factors and MTT metabolization was measured at 24 (A), 48 (B), 72 (C) or 96 (D) hours. Mean absorbance values of untreated samples were taken as 100% and then mean values referred to that. Data are represented as mean ± SD of quadruplicates of an experiment that was repeated at least twice.

The *in vitro* action of OOS was initially evaluated on several AML cell lines. With this purpose, the AML cell lines HEL, HL60 and KG1 were grown in the presence of increasing doses of OOS diluted into the culture medium and their proliferation was evaluated in MTT assays at different days of treatment. OOS decreased MTT metabolization in the three cell lines tested (Figure 1). Moreover, this effect was more evident at longer incubation times (Figure 1D). The IC_50_ values ranged from 1/10-1/15 at 24 hours to up to 1/137 at 96 hours for the HEL cell line. Comparing the three cell lines tested, HEL cells were the most sensitive to the action of OOS at the different times and concentrations tested.

### Effect of OOS in combination with anti-AML treatments

**Figure 2:**
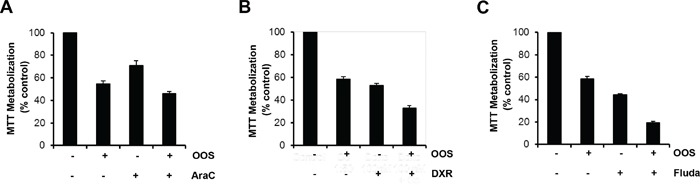
OOS is more effective in combination with standard of care treatments The effect of OOS alone (1:50) or in combination with Ara C (250 nM, A), Doxorubicin (DXR, 100 nM, B) or Fludarabine (Fluda, 250 nM, C) were determined in MTT assays. The mean absorbance values of untreated samples from each were considered as 100%. Data are represented as mean ± SD of quadruplicates of an experiment that was repeated at least twice.

In most of the cases, the success of antitumor therapies is based on the combination of different agents [15]. For this reason we wondered if the combination of OOS with drugs commonly used in the AML clinic could improve their antitumor effect. Thus, OOS was administered *in vitro* to HEL cells alone (dilution factor 1/50), or in combination with Ara C (250 nM), Doxorubicin (DXR, 100 nM) or Fludarabine (Fluda, 250 nM). In all the three cases the combination was more effective inhibiting the cell growth than the independent treatment with any of the agents (Figure 2).

### *In vivo* efficacy of OOS in AML murine models

**Figure 3:**
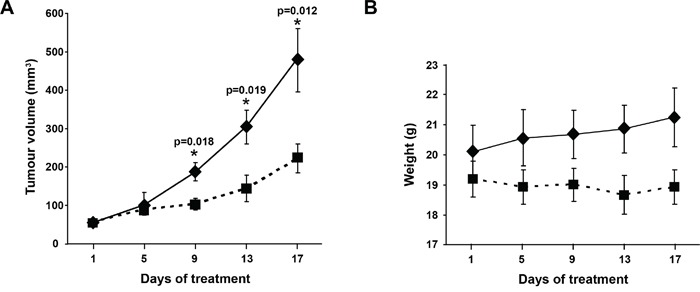
Efficacy of OOS on AML models *in vivo* (A) OOS interferes with tumor growth. Female CB17-SCID athymic mice were injected with HEL cells. When tumors became palpable and were growing, they were randomized to different groups that were orally treated 5 days per week (Monday to Friday) with 100μl OOS/animal (■) or vehicle alone (water,♦), and tumor volumes were measured twice a week. Data are represented as mean tumor volume ± SEM of the animals on each group. (B) Effect of OOS on animal weight. Statistical significant differences are shown (*p<0.05).

Given the action of OOS *in vitro*, we explored whether this *in vitro* effect could translate *in vivo*. With that purpose we used a xenograft murine model to determine the effect of OOS treatment once the tumors have already been established. CB17-SCID athymic mice were orthotopically injected with the AML cell line HEL and when tumors were palpable, animals were randomized to 4 different groups to orally receive water or increasing amounts of OOS: 100, 200 or 300 μl OOS per 20 g of animal weight. Initially, tumors included in the four groups had a similar volume (53±4.9; 56±5.4; 53±4.9 and 52±3.1 mm^3^, mean ± SEM, respectively). After 17 days of daily oral treatment with OOS (Monday to Friday), a decrease in the growth of the tumors was observed (figure 3A and data not shown). Thus the mean tumor volume of control mice was 484±75.8 mm^3^, as compared with 222±37.3, 207±66.5 or 232±61.9 mm^3^ corresponding to 100, 200 or 300 μl OOS, respectively. The mean body weight of control or treated mice did not decrease throughout the duration of the experiment (figure 3B and data not shown), indicative of no significant toxicity of OOS in the different experimental conditions. Thus, at the day of sacrifice, mean body weight in control mice was 21.3±1 grams compared to 18.9±0.6, 19.5±0.5 and 19.5±0.3 grams in the different OOS treated groups.

### Effect of OOS on circulating cytokines

**Figure 4:**
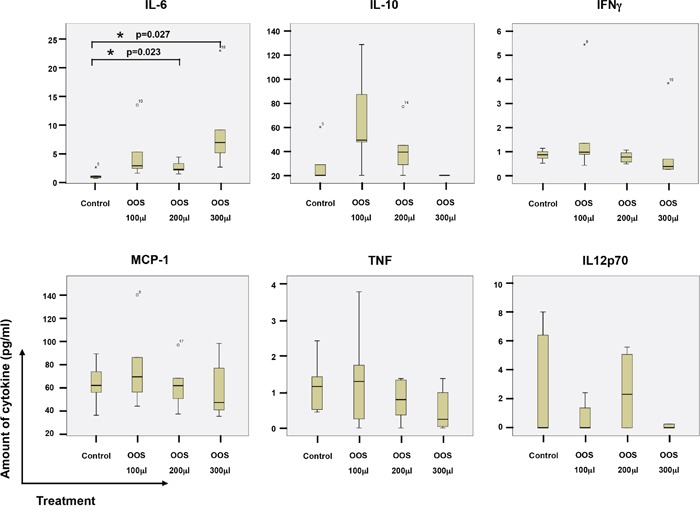
Action of OOS on immune system cytokines At the time of sacrifice, blood from the animals under the different treatments was collected by intracardiac injection, and sera clarified and frozen. The amount of the indicated cytokines was determined by flow cytometry using a BD Cytometric Bead Array as described in the materials and methods section. The amount for each molecule and experimental condition are shown in the corresponding boxplots, as indicated. Statistical significant differences are shown (*p<0.05).

As mentioned above, some components of OOS such as glycyrrhizinic acid exert immunomodulatory actions [12]. Because of this and the relevant role of the immune system in the control of tumor progression, we explored whether this formulation could affect immune system properties through the measurement of cytokine levels in mice circulating blood. Thus, the levels of IL-6, IL-10, IFNγ, MCP-1, TNFα and IL12p70 were measured in the sera collected from the animals at the time of sacrifice. An increase in the level of IL6 was observed as the amount of OOS used in the treatment rose. In fact, significant differences for this cytokine were observed between the control group and those treated with 200 or 300 μl OOS (p=0,023 or p=0,027 respectively) (Figure 4). The same tendency was observed for the 100 μl group but in this case differences were not statistically significant (p=0.079), probably due to the small number of animals. No major changes were observed in the amount of the other cytokines tested (Figure 4). Moreover, there was no correlation between those levels and the amount of OOS used for the treatment.

### Effect of OOS cell proliferation and apoptosis *in vivo*

**Figure 5:**
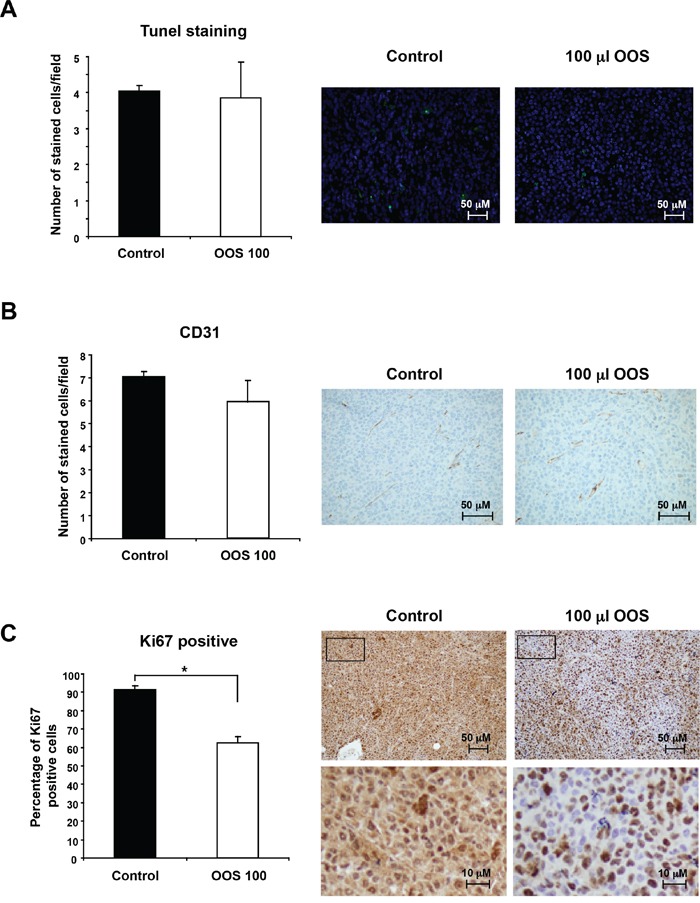
OOS induces a decrease in tumor proliferation (A) OOS does not induce apoptotic cell death on AML tumors. For each experimental condition, two tumors were randomly processed for IHQ analysis and apoptotic cells were detected by tunnel staining. The number of apoptotic cells/field was quantified for each condition and its mean number ± SD is shown in the graph. Pictures of representative fields stained for this marker are shown on the right. (B) Similarly the number or endothelial cells was measured by CD31 staining. (C) Besides, to establish the proliferative status of the tumors, Ki67 marker was used. Pictures of representative fields stained for Ki-67 are shown on the right upper row and lower pictures show part of them (inset) at higher magnification. Statistical significant differences are shown (*p<0.05).

Because OOS caused a decrease in tumor growth when compared to untreated controls, we questioned if that effect was caused by an increased cell death, a lower rate of proliferation or vascularization or a combination of any of the above. When sections of the tumors were stained to identify apoptotic cells, using the conventional TUNEL staining technique, no differences in the number of apoptotic cells were observed between the different groups (Figure 5A).

On the other hand, when tumors develop, a blood supply is necessary for them to grow beyond a few millimeters in size [3, 16]. For this reason, the presence of blood vessels in the tumor can be considered an indicator of tumor activity and progression, and such presence can be assessed by staining endothelial cells with the specific marker CD31 [17, 18]. Even though OOS treated tumors grew slower, no differences in the number of cells stained for this marker were observed among the different groups (Figure 5B).

Finally, the expression of human Ki-67, a protein associated with cell proliferation, was evaluated. To verify if OOS treatment induced changes in the proliferation of the tumors, staining for this marker was carried out. Treatment with OOS induced an evident decrease in the percentage of Ki-67 positive cells (Figure 5C). Moreover, such a decrease was statistically significant when compared with control untreated tumors (Figure 5C).

**Figure 6:**
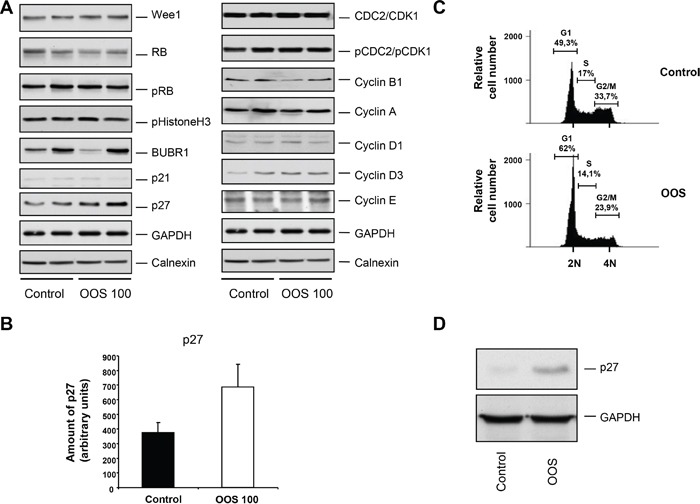
OOS causes accumulation of p27 and G1 arrest (A) Quantitation of the *in vivo* levels of cell cycle related proteins. The amount of the indicated proteins in the tumors was assessed by conventional WB. (B) The amount of p27 was quantified using the NIH image software, and the mean ± SD of such amount is represented in the graph. (C) Cell cycle analysis of HEL cells treated for 24 hours with OOS (1/50 dilution). The percentage of cells in the different phases of the cell cycle is shown. (D) Action of OOS (1/50) on p27 levels. HEL cells were treated for 24 hours with OOS and cell extracts prepared to analyze p27 by WB. GAPDH was used as a loading control.

To further investigate the mechanism by which these tumors exhibited a decrease in proliferation, the amount and status of several proteins involved in cell cycle progression was evaluated by Western Blot analysis. This panel of proteins included markers of the different phases of the cell cycle, such as Wee1, BUBR1, pHistoneH3, pRB or p21 and p27, or different cyclins and CDKs, (Figure 6A). Representative tumors were chosen and lysed to prepare protein extracts as described in the materials and methods section. Proteins were separated in SDS-PAGE gels and the membranes probed with the indicated antibodies. For most of the proteins analyzed, no mayor differences in the control versus treated tumors were observed. Interestingly the levels of the cell cycle inhibitor p27 were upregulated in tumors from animals that had been treated with OOS (Figure 6A and B). p27 is an inhibitor of the Cyclin E/Cdk2 and Cyclin D/Cdk4 complexes, and its higher levels would slow down cell cycle progression, mainly blocking the cell cycle progression through the G1 phase. To analyze this hypothesis, HEL cells were *in vitro* treated with OOS, and the cell cycle profile was analyzed. In those conditions, an accumulation in the G1-phase population and a correlative G2-M decrease was detected (Figure 6C). Moreover, when p27 levels were analyzed *in vitro* in those conditions, a clear increase in this protein was observed in OOS treated cells (Figure 6D).

### Transcriptional effect of OOS *in vivo*

**Figure 7:**
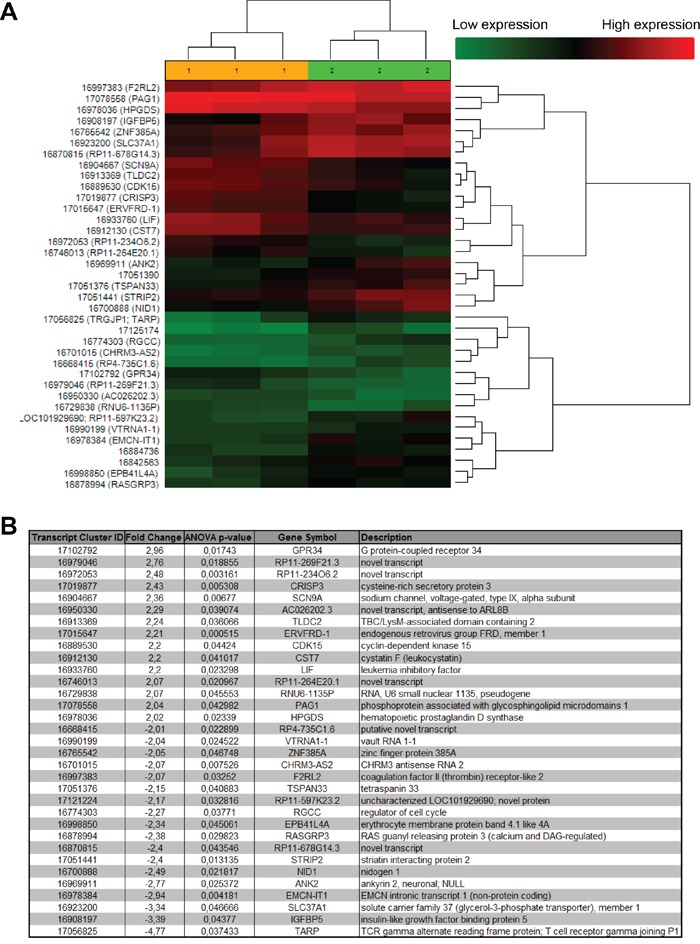
*In vivo* effect of OOS on gene expression profiles (A) Hierarchical clustering of the 6 tumors and the 37 genes deregulated after OOS treatment. Each row represents a gene and each column represents a tumor (1- control, 2- OOS treated). The expression level of each gene in each tumor is relative to its medium abundance across all the tumors and is depicted according to the color scale shown. Red and green indicate high or low expression levels, respectively. (B) The gene expression profile of three control tumors was compared to that of three tumors treated with 100 μl of OOS. A list with those genes whose expression changed above 2 times with the treatment is shown in the figure.

In order to better understand the mechanisms by which OOS executes its action, we investigated the changes in gene expression profiles derived from the *in vivo* treatment with OOS. Thus, gene expression analyses were performed in triplicates of the tumors from control untreated animals and compared with those from animals treated with OOS that had exhibited a slower tumor growth. Unsupervised clustering of the gene expression data associated samples into two groups, control and OOS-treated, indicating that treatment with OOS was able to sufficiently change the transcriptome to make treated samples to diverge from the untreated control tumors (Figure 7A). Quantitative comparison of both conditions defined 150 (fold change of 1.5, Supplementary Table 1) deregulated genes. Of them, 33 genes were deregulated more than two-fold (Figure 7B). Within those genes the most downregulated gene after OOS treatment was the G protein coupled receptor 34 (GPR34) that was 2.96 times more expressed in the control condition. On the other hand the T cell receptor TARP was the most upregulated gene (4.77 times) after OOS treatment. Besides within the deregulated genes there are other genes related to membrane receptors or ionic channels, such as SNC9A or TSPAN33. Pathway analyses defined routes particularly affected by the treatment with OOS. A complete listing of the results of pathway analyses is shown in Supplementary figure 1. Among the routes affected were genes involved in cell cycle regulation, as CDK15 or RGCC, or other genes involved in EGFR, IGF or TGF beta signaling or the leukemia inhibitory factor (LIF).

## DISCUSSION

In this study we have evaluated the antitumoral effect of OOS in AML, using both *in vitro* and *in vivo* models. OOS induced a clear decrease in MTT metabolization, indicative of antitumoral effect, in the different AML derived cell lines tested, and such reduction was both time and dose dependent. Moreover, OOS reduced tumor progression *in vivo* in mice implanted with the cell line HEL, which was the most sensitive cell line to OOS *in vitro*, and was used to generate xenograft models. OOS treatment slowed down tumor growth without apparent toxicity, since the weights of the control and treated mice were analogous, and no detectable changes in their behavior were observed. Thus, OOS demonstrated a clear effect on AML tumor progression.

Since OOS is a nutritional supplement that includes recognized anti-oxidants in its formulation, chances are that these agents would also have a preventive action *in vivo*. In fact this action has been recently described for other models such as breast cancer models in which OOS has antitumor effects both preventive, and once the tumors have been established [14]. If OOS could also have such a preventive action on AML models should be similarly tested.

To gain insights into the mechanisms leading to the reduction of tumor burden *in vivo*, several studies were carried out. To evaluate if it was due to a reduction in cell duplication, an increase in cell death or both, sections of the tumors were stained with appropriate markers. There was no effect of OOS on cell death since no differences on the number of cells stained by TUNEL were observed when compared to tumors from untreated animals. Similarly, no differences on the vascularization marker CD31 were found. It was only when the proliferation marker Ki67 was used to stain the sections when significant differences were found. Tumors from animals treated with OOS exhibited a lower percentage of cells stained with this marker, pointing to a decrease in cell proliferation in those conditions. To better characterize this effect, the amount and status of different cell cycle markers were determined by WB. There were no differences in the CDKs or cyclins tested. The main differences were observed in the levels of p27^Kip1^. This is an inhibitor of cell cycle progression encoded by the CDKN1B gene and whose presence prevents the activation of the cyclin E-CDK2 and cyclin D-CDK4 complexes, and thus blocks the cell cycle at the G1 phase [19]. In fact p27 has been described to play a role in carcinogenesis, and could probably be used as a prognostic factor in different tumors, as well as an indicator of response to therapies [20]. Besides these data corroborate those found for the mechanism of action of OOS on breast cancer models. In that model OOS induces both cell death and cell cycle arrest in G1, this latter due to an increase in p27 levels, concomitant with a decrease in cyclin D1 [14].

So far, the general mechanism of OOS action is not clear, although it points to inhibitory effects on cell cycle progression. The analysis of the genes whose expression changed after OOS treatment identified some involved in cell cycle regulation and progression such as CDK15 or RGCC. The function of these proteins as cell cycle regulators is not fully known and its potential role in AML should be carefully analyzed in this context. Interestingly, OOS caused a decrease in the amount of LIF. This polypeptide acts by binding to the LIF receptors and gp130, and such binding triggers the activation of several signaling pathways, including the JAK/STAT, MAPK, and PI3K routes. In different cell types, LIF may exert opposite effects including stimulation or inhibition of cell proliferation [21].

One of the characteristics of AML is the replacement of normal blood cells with leukemic cells, which makes the patients more susceptible to infections [22, 23]. In our study OOS seems to stimulate the immune system through the production of IL6. Thus, if OOS not only blocks the proliferation of AML cells, but also stimulates the immune system, patients could benefit from this double advantage.

Most antitumor therapies rely on the combination of agents to increase the antitumor action of the individual agents [15]. In this respect, when OOS was used in combination with AML standard of care treatments, a potentiation of their antiproliferative effect was found. This observation would establish the rationale to test the therapeutic potential of OOS in AML patients alone or in combination with other antitumoral agents. Especial attention should be paid to patients with relapsed AML, since in them the therapeutic options are very limited and in most of the cases, only palliative care can be offered [24]. In this group of patients OOS addition to gold standard therapies could augment their antitumoral properties and be, therefore, an interesting therapeutic option.

## MATERIALS AND METHODS

### Reagents and antibodies

Cell culture media, 3-(4,5-dimethylthiazol-2-yl)-2,5-diphenyltetrazolium bromide (MTT), hematoxylin and eosin were purchased from Sigma. Foetal bovine serum (FBS) and antibiotics were from Life Technologies and Immobilon P (PVDF) membranes from Millipore. OOS was provided by Catalysis, S.L. (Madrid, Spain). The Cytometric Bead Array Mouse Inflammation kit was purchased to BD biosciences. Other generic chemicals were purchased from Sigma Chemical Co., Roche Biochemicals or Merck.

The origin of the different antibodies used in the Western blotting analyses were as follows: the anti-GAPDH, anti-p21, anti-Wee1, anti-CDC2/CDK1, anti-pCDC2, anti-Cyclin B and anti-Cyclin D1 antibodies were purchased from Santa Cruz Biotechnology; the anti-BUBR1, anti-Rb, anti-Cyclin A, anti-Cyclin D3 and anti-Cyclin E from BD Biosciences; the anti-p27 and the anti-pRb^S780^ from Cell Signaling technology; the anti-Calnexin from Stressgen and the anti-pHistone H3 from Millipore. The horseradish peroxidase-conjugated secondary antibodies were from Bio-Rad.

### Cell culture, cell cycle and cell proliferation measurement

AML cell lines were grown in RPMI 1640 medium supplemented with 10% FBS and antibiotics. All the cell lines were cultured at 37°C in a humidified atmosphere in the presence of 5% CO_2_-95% air. The cell lines were obtained from the American Type Culture Collection, Cell Biology Collection (Manassas, VA), or have already been described [25].

For cell cycle analysis by flow cytometry, ethanol fixed cells were stained with 5 μg/ml propidium iodide (PI) and 250 μg DNAse-free RNAse. A total of 50,000 cells were acquired in the propidium iodide gate by using a BD Accuri^TM^ C6 flow cytometer and the C6 software (BD Biosciences).

Cell proliferation of AML cells was examined using a modified MTT metabolization assay [26]. Briefly, cells were plated in triplicates and, on the day of the experiment, MTT was added to the wells at 0.5 mg/ml and incubated at 37°C for 4 hours. The MTT-formazan crystals were dissolved in isopropanol-HCl and the absorbance of the samples was recorded at 570 nm using a Tecan spectrophotometer with the X-Fluor 4 software. At least three wells were analyzed for each condition, and the results are presented as the mean +/− SD of a representative experiment repeated at least twice.

### In vivo experiments

For animal studies, twenty-four 7-week-old female athymic mice (CB17-SCID) were purchased from Charles River Laboratories (Wilmington, MA), and kept in pathogen free housing at our Institutional Animal Care Facility. One week later, 3×10^6^ HEL cells resuspended in 50 μl of RPMI-1640 and 50 μl of Matrigel were subcutaneously injected into the right caudal flank of each animal. When tumors became palpable, mice were randomized into four groups (n = 6 per group), that were administered the different treatments: control group (receiving the vehicle alone) or various concentrations of OOS, including 100 μl, 200 μl, or 300 μl per mouse (20 g). Treatments were administered for 17 days with a daily schedule (Monday to Friday) by oral gabage. Mice were weighted and tumors measured twice a week with a digital caliper (Proinsa, Vitoria, Spain). Tumor volumes were calculated using the following formula: V = (L/2) x (W/2)^2^ x 4/3 x π, where V = volume (cubic mm), L = length (mm) and W = width (mm). At the time of sacrifice, tumor tissue was resected and immediately frozen at −80°C. Besides, blood was extracted by intracardiac injection, and the sera collected and frozen at −80°C for further analyses.

### Protein extraction and Western blotting (WB)

Frozen mice tumor samples were minced, washed with phosphate-buffered saline (PBS), and homogenized in ice-cold lysis buffer [26], that had been supplemented with 10X protease inhibitors using a tight-fitting Dounce homogenizer. This homogenate was centrifuged at 4°C, and the supernatants were transferred to new tubes for protein concentration measurement by Bradford assay. Boiled samples were separated on 6%–15% SDS-PAGE gels, depending on the molecular weight of the proteins to be analyzed, and after electrophoresis, proteins in gels were transferred to PVDF membranes and WB performed as previously described [26]. Bands were visualized by using ECL Western Blotting Detection System (GE Healthcare, Buckinghamshire, United Kingdom). Similarly, protein extracts from OOS-treated cells were prepared and analyzed.

### Analysis of circulating cytokines

To determine the amount of different cytokines present in mice serum samples, the BD^TM^ CBA Mouse Inflammation kit was used, following the providers' instructions. Briefly, sera to be tested were thawn on ice while in parallel the capture beads specific for the cytokines were properly mixed and 50 μl added to the assay tubes. On top of them, either 50 μl of the unknown samples, or the mouse inflammation standards previously prepared by serial dilution were added and incubated in the dark for 2 hours at room temperature. One ml wash buffer was added and the beads spun down. After aspirating the supernatant, pellets were resuspended in 300 μl wash buffer and samples analyzed in a BD Accuri^TM^ C6 cytometer. Data were further analyzed using the BD FCAP Array^TM^ software version 3.0.

### Histological and immunohistochemistry (IHC) analyses

Representative tumor areas were fixed in formalin, paraffin embedded, cut in 2- to 3-μm sections, and either stained with hematoxylin and eosin or prepared for IHC, that was performed as previously described [27]. Thus, 2 cell conditioning periods of 8 min at 95°C and 4 min at 100°C on hot plate using TRIS-EDTA (pH=8) buffer were performed on previously dewaxed formalin-fixed paraffin-embedded sections. Sections were then incubated with the anti-Ki67 or anti-CD31 antibodies, and the staining was performed with the IHC 3,3′-diaminobenzidine MAPO system (Ventana Medical Systems). For the TUNEL staining the In Situ Cell Death Detection Kit (Roche Mannheim, Germany) was used, and counterstained with 4′,6-diamino-2-phenylindole. Results were evaluated blinded to clinicopathologic and molecular data. The number and intensity of immunoreactive cells were evaluated in at least ten randomly selected fields. These procedures were done by independent personnel of the pathology unit of our center. Conflict measurements were solved by consensus.

### RNA isolation, cDNA synthesis and microarray hybridization and analysis

After thawing, tumors were excised and lysed in Trizol Reagent (Life Technologies), according to the manufacturer' instructions. Briefly, tumors were homogenized (Dispomix, L&M Biotech, Holly Springs, NC, USA) and incubated in the Trizol solution for 2 minutes at room temperature, before the addition of chloroform. Tubes were vigorously shaken and the different phases separated by centrifugation. The upper, aqueous phase was recovered and the RNA present precipitated with isopropyl alcohol. Once washed in 70% ethanol the resultant RNA was column-purified (Qiagen, RNeasy mini kit) and its integrity was assessed (Agilent 2100 Bioanalyzer, Agilent). Biotinylated complementary RNA was then synthesized (Enzo Life Sciences) and hybridized to HG-U133 plus 2.0 GeneChip oligonucleotide arrays (Affymetrix). Quantitation of fluorescence intensities of probesets was done using the GenArray Scanner (Hewlett Packard). Unprocessed files were normalized using the RMA algorithm implemented in the Affimetrix Expression Console. Differentially expressed genes were identified using significant analysis of microarrays, selecting all genes with a value of Q≤0.05. Microarray data are now available through the GEO repository database (reference GSE75174).

### Statistical analysis

Each condition was analyzed in triplicate or quadruplicate and data are presented as the mean ± SD of at least three independent experiments. Comparisons of continuous variables between two groups were performed using two-sided Student's *t* test. Differences were considered to be statistically significant when *P* values were less than 0.05.

## SUPPLEMENTARY TABLE AND FIGURE




