# Supplementary material for: Antitumoral effect of Ocoxin on acute myeloid leukemia
Source: Oncotarget. 2016 Jan 9;7(5):6231–42. doi: 10.18632/oncotarget.6862 (PMC4868752; doi:10.18632/oncotarget.6862)
Supplement: Supplementary file 1 [file oncotarget-07-6231-s001.pdf]

SUPPLEMENTARY TABLE AND FIGURE

Supplementary Table S1: In vivo effect of OOS on gene expression profiles. The gene expression profile of three control tumors was compared to that of three tumors treated with 100 µl of OOS. A list with those genes whose expression changed above 1.5 times with the treatment is shown in the table.

See Supplentary table 1

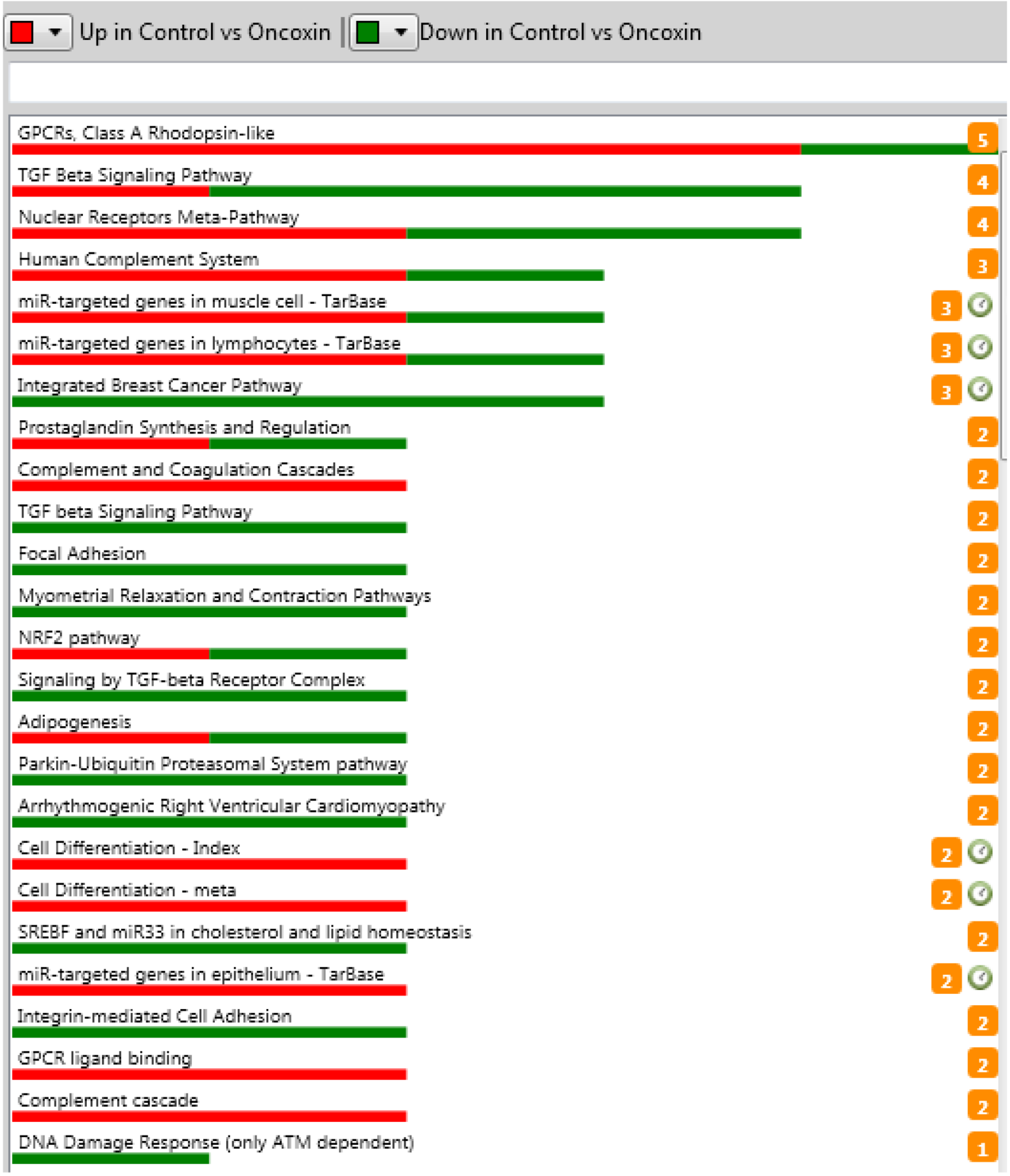
Up in Control vs Oncoxin
Down in Control vs Oncoxin
GPCRs, Class A Rhodopsin-like
5
TGF Beta Signaling Pathway
4
Nuclear Receptors Meta-Pathway
4
Human Complement System
3
miR-targeted genes in muscle cell - TarBase
3
miR-targeted genes in lymphocytes - TarBase
3
Integrated Breast Cancer Pathway
3
Prostaglandin Synthesis and Regulation
2
Complement and Coagulation Cascades
2
TGF beta Signaling Pathway
2
Focal Adhesion
2
Myometrial Relaxation and Contraction Pathways
2
NRF2 pathway
2
Signaling by TGF-beta Receptor Complex
2
Adipogenesis
2
Parkin-Ubiquitin Proteasomal System pathway
2
Arrhythmogenic Right Ventricular Cardiomyopathy
2
Cell Differentiation - Index
2
Cell Differentiation - meta
2
SREBF and miR33 in cholesterol and lipid homeostasis
2
miR-targeted genes in epithelium - TarBase
2
Integrin-mediated Cell Adhesion
2
GPCR ligand binding
2
Complement cascade
2
DNA Damage Response (only ATM dependent)
1

(*Continued*)

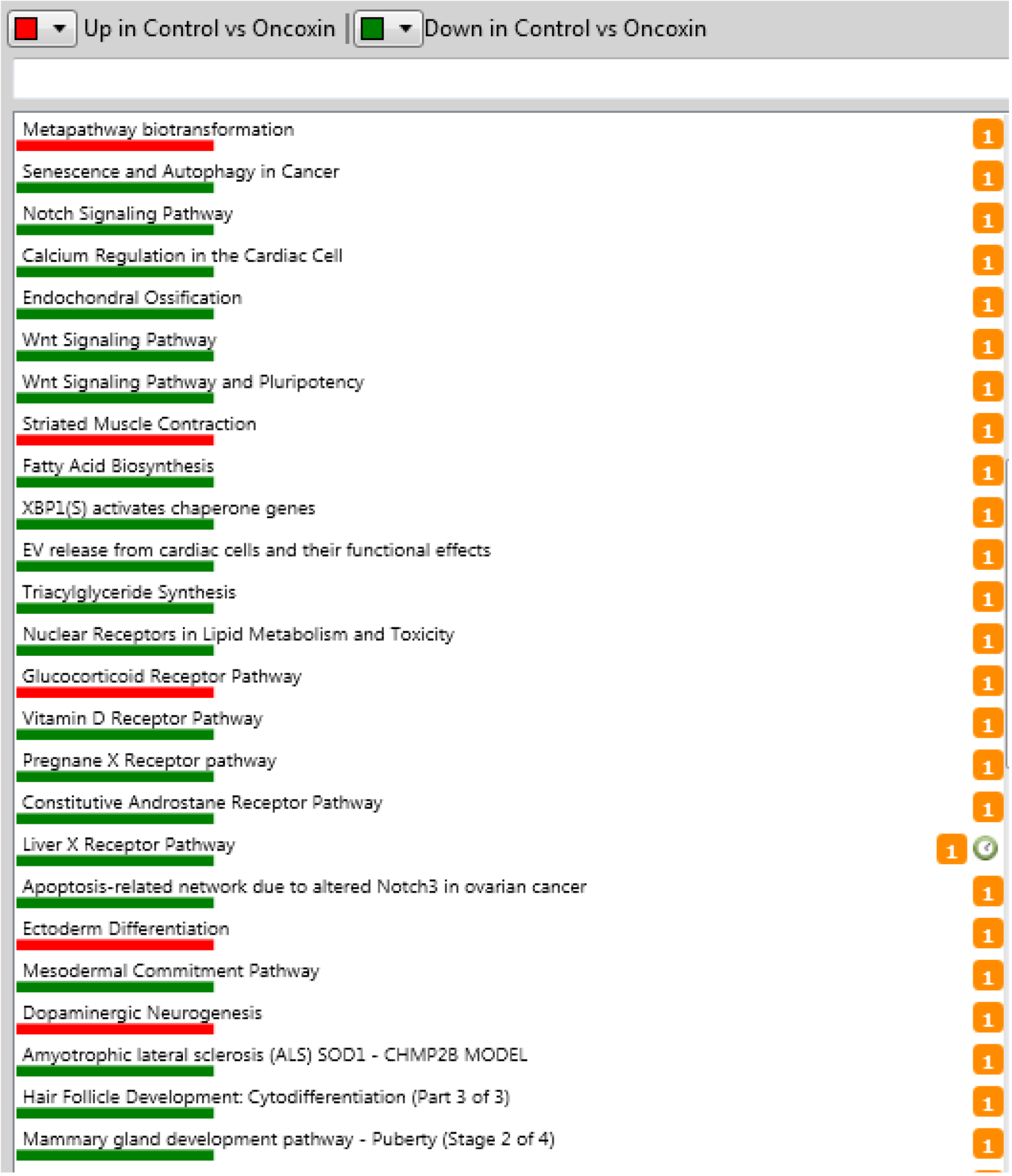
Up in Control vs Oncoxin
Down in Control vs Oncoxin
Metapathway biotransformation
Senescence and Autophagy in Cancer
Notch Signaling Pathway
Calcium Regulation in the Cardiac Cell
Endochondral Ossification
Wnt Signaling Pathway
Wnt Signaling Pathway and Pluripotency
Striated Muscle Contraction
Fatty Acid Biosynthesis
XBP1(S) activates chaperone genes
EV release from cardiac cells and their functional effects
Triacylglyceride Synthesis
Nuclear Receptors in Lipid Metabolism and Toxicity
Glucocorticoid Receptor Pathway
Vitamin D Receptor Pathway
Pregnane X Receptor pathway
Constitutive Androstane Receptor Pathway
Liver X Receptor Pathway
Apoptosis-related network due to altered Notch3 in ovarian cancer
Ectoderm Differentiation
Mesodermal Commitment Pathway
Dopaminergic Neurogenesis
Amyotrophic lateral sclerosis (ALS) SOD1 - CHMP2B MODEL
Hair Follicle Development: Cytodifferentiation (Part 3 of 3)
Mammary gland development pathway - Puberty (Stage 2 of 4)

(*Continued*)

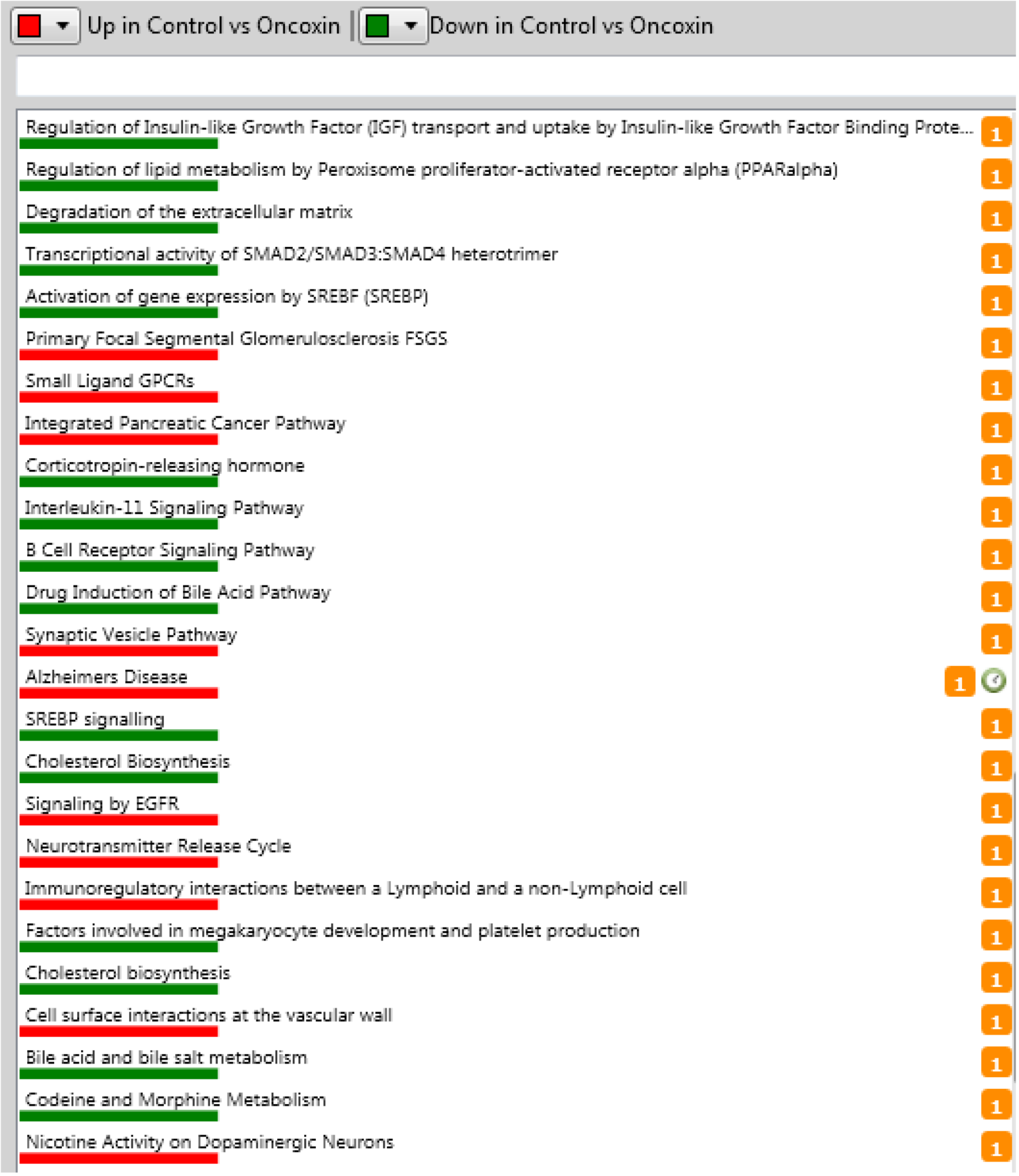
Up in Control vs Oncoxin
Down in Control vs Oncoxin
Regulation of Insulin-like Growth Factor (IGF) transport and uptake by Insulin-like Growth Factor Binding Prote...
Regulation of lipid metabolism by Peroxisome proliferator-activated receptor alpha (PPARalpha)
Degradation of the extracellular matrix
Transcriptional activity of SMAD2/SMAD3:SMAD4 heterotrimer
Activation of gene expression by SREBF (SREBP)
Primary Focal Segmental Glomerulosclerosis FSGS
Small Ligand GPCRs
Integrated Pancreatic Cancer Pathway
Corticotropin-releasing hormone
Interleukin-11 Signaling Pathway
B Cell Receptor Signaling Pathway
Drug Induction of Bile Acid Pathway
Synaptic Vesicle Pathway
Alzheimers Disease
SREBP signalling
Cholesterol Biosynthesis
Signaling by EGFR
Neurotransmitter Release Cycle
Immunoregulatory interactions between a Lymphoid and a non-Lymphoid cell
Factors involved in megakaryocyte development and platelet production
Cholesterol biosynthesis
Cell surface interactions at the vascular wall
Bile acid and bile salt metabolism
Codeine and Morphine Metabolism
Nicotine Activity on Dopaminergic Neurons

(Continued)

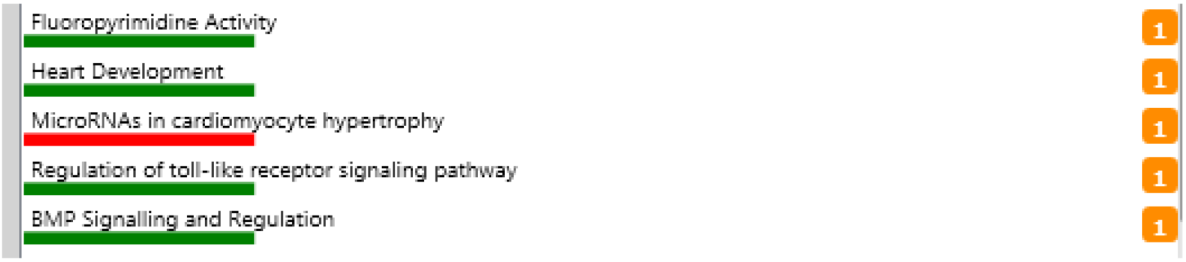

Supplementary Figure S1: Pathways in vivo deregulated after OOS treatment. Genes found to be deregulated al least 1.5 times in the Affimetrix Expression Console were analyzed with the same software to evaluate the pathways in which gene expression is altered. Below each altered pathway, genes upregulated in the control condition (red) or after OOS treatment (green), are shown. Besides, the total number of genes deregulated in a given pathway are shown in an orange box at the end of each line.
